# Supplementary material for: Molecular Basis of the Anticancer and Antibacterial Properties of CecropinXJ Peptide: An In Silico Study
Source: Int J Mol Sci. 2021 Jan 12;22(2):691. doi: 10.3390/ijms22020691 (PMC7826669; doi:10.3390/ijms22020691)
Supplement: Supplementary file 1 [file ijms-22-00691-s001.pdf]

Supplementary Materials

Molecular basis of the anticancer and antibacterial properties of CecropinXJ peptide: An In Silico Study

Francisco Ramos-Martín and Nicola D'Amelio

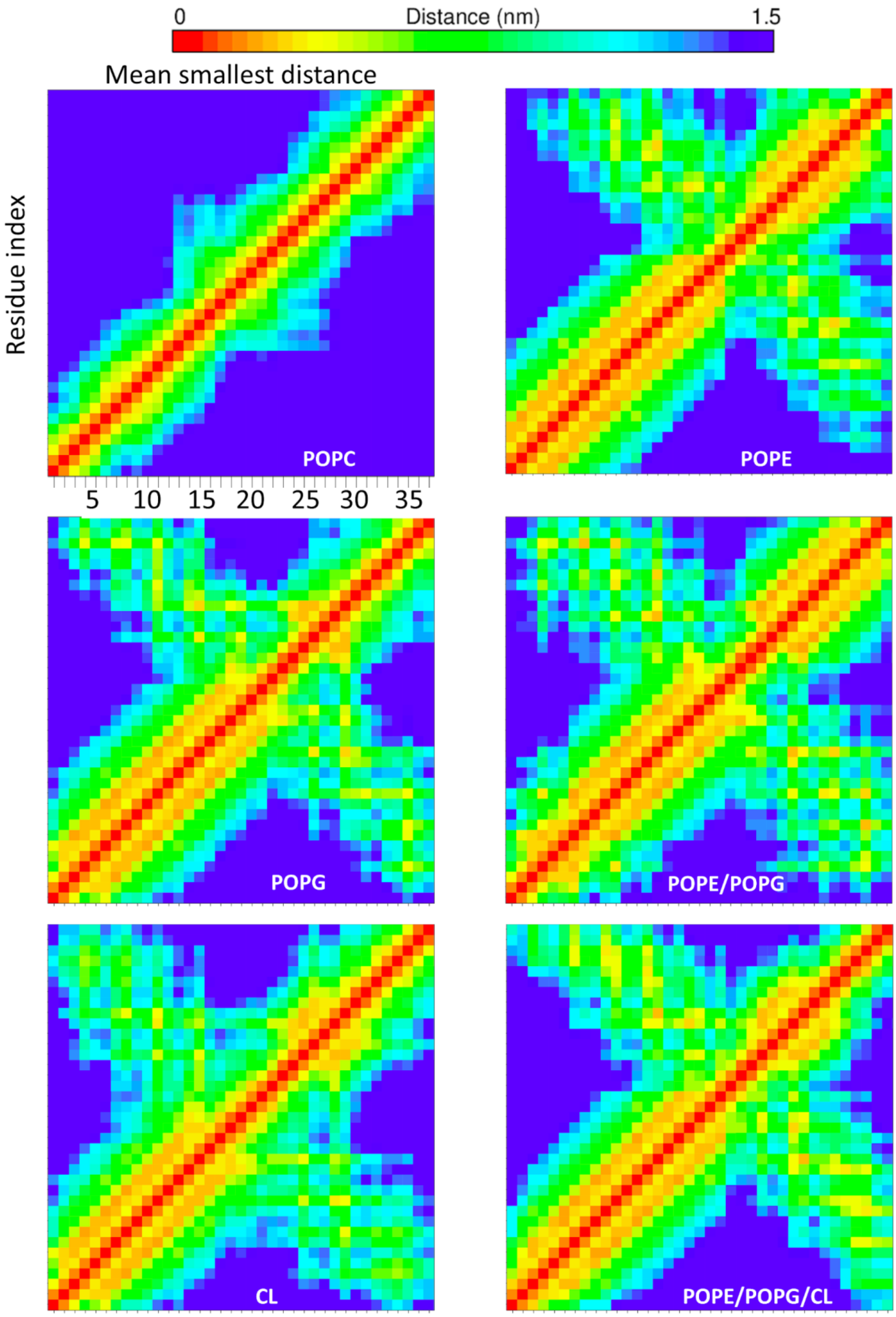

Figure S1. Contact maps comparing CXJ conformation with POPC and bacterial mimic membranes (POPE, POPG, POPE/POPG, CL and POPE/POPG/CL).

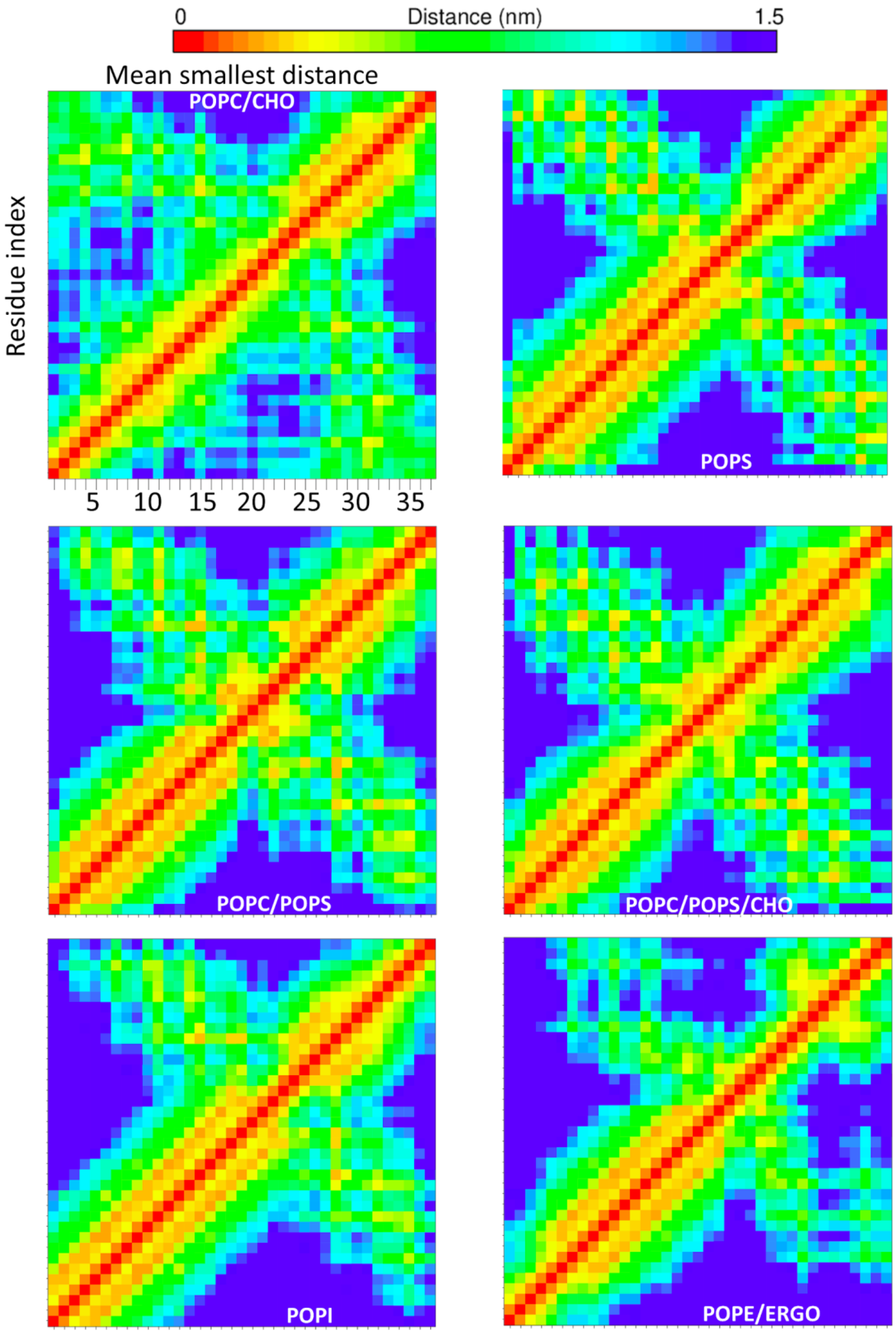

Figure S2. Contact maps comparing CXJ conformation with POPC/CHO, POPI, POPE/ERGO and POPS-containing membranes.

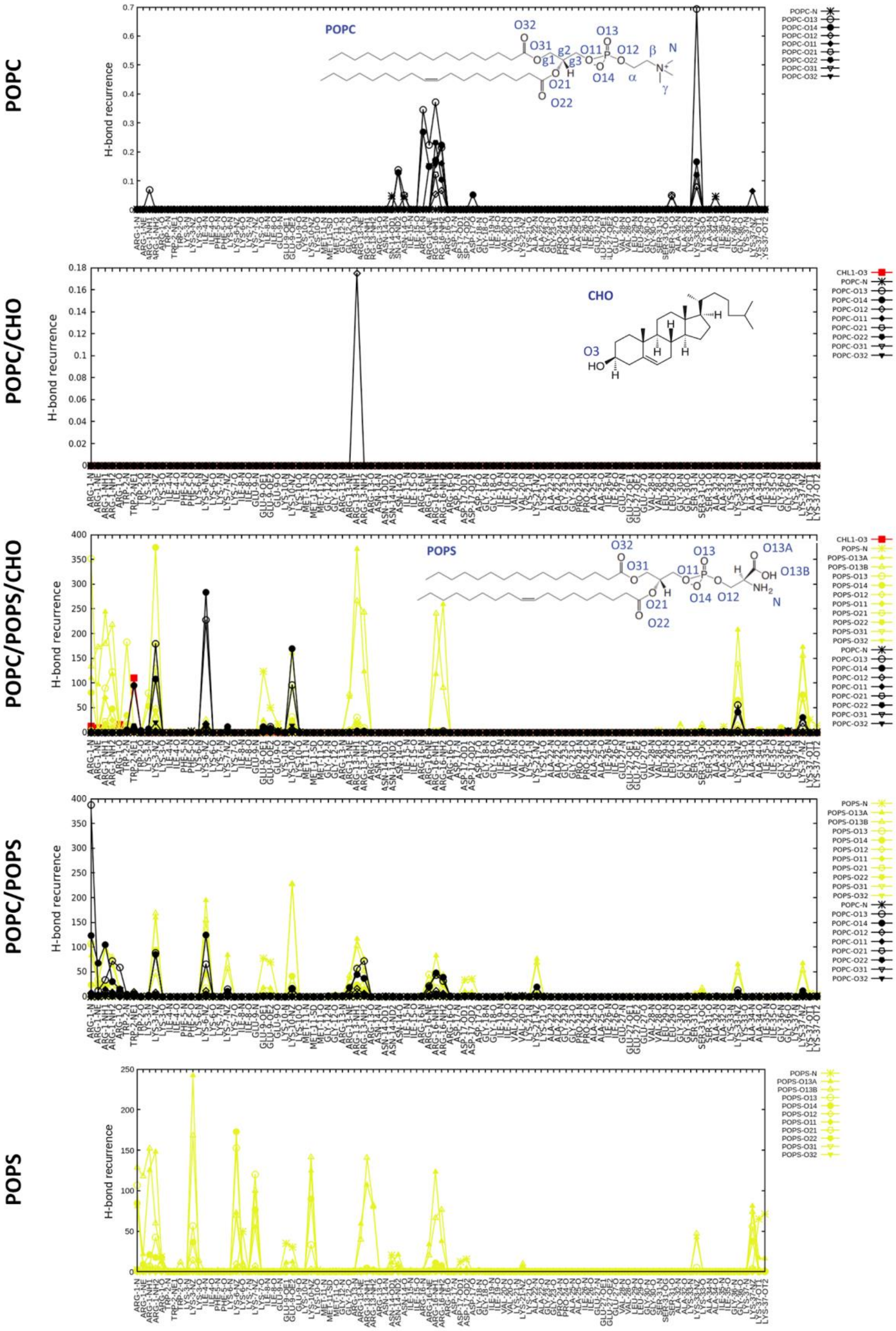

Figure S3. Occurence of polar atom contacts (H-bonds and salt bridges) between CXJ peptide and various membrane bilayers calculated along MD simulation trajectories.

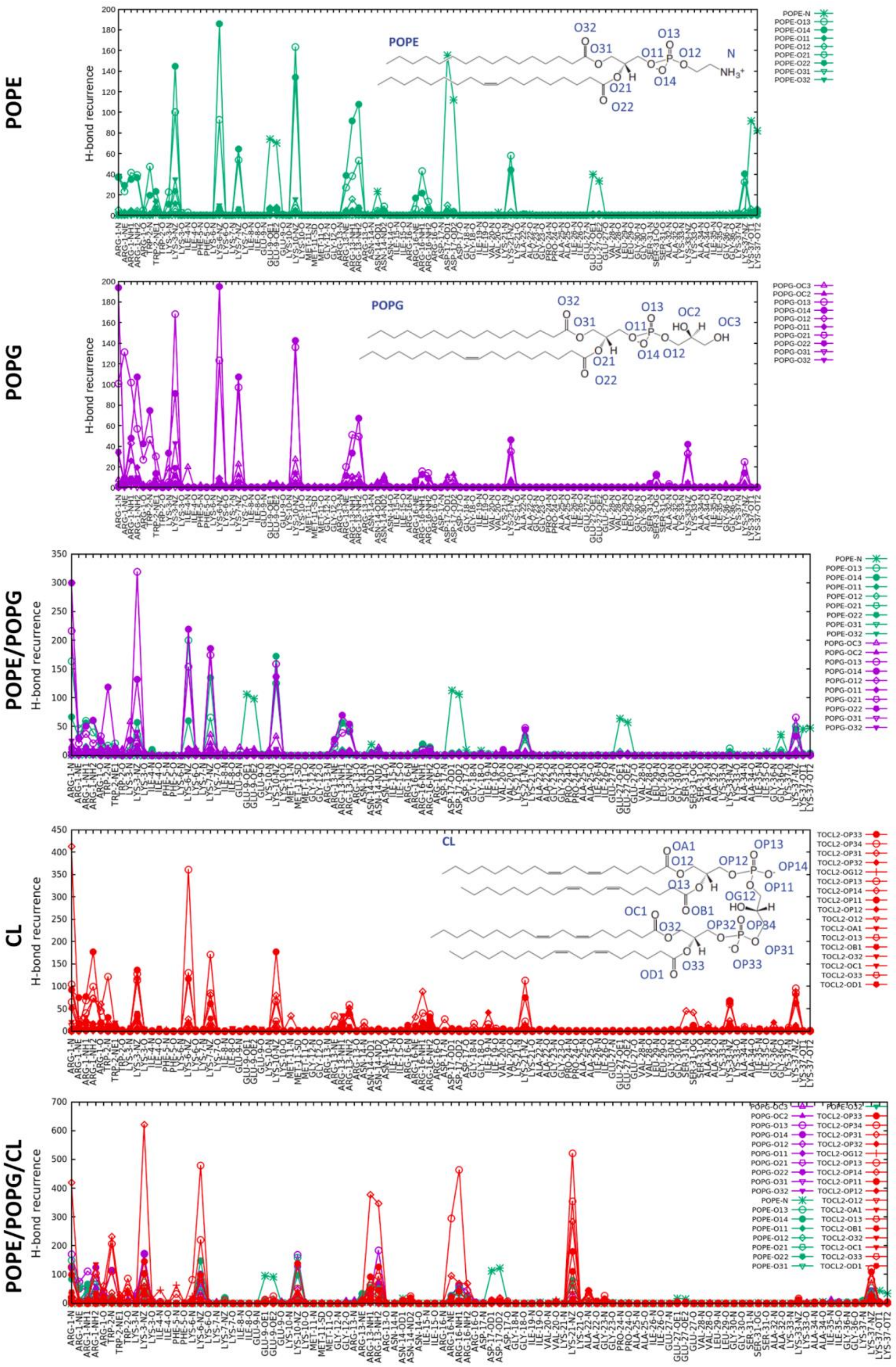

Figure S4. Occurence of polar atom contacts (H-bonds and salt bridges) between CXJ peptide and various membrane bilayers calculated along MD simulation trajectories. TOCL2 refers to CL.

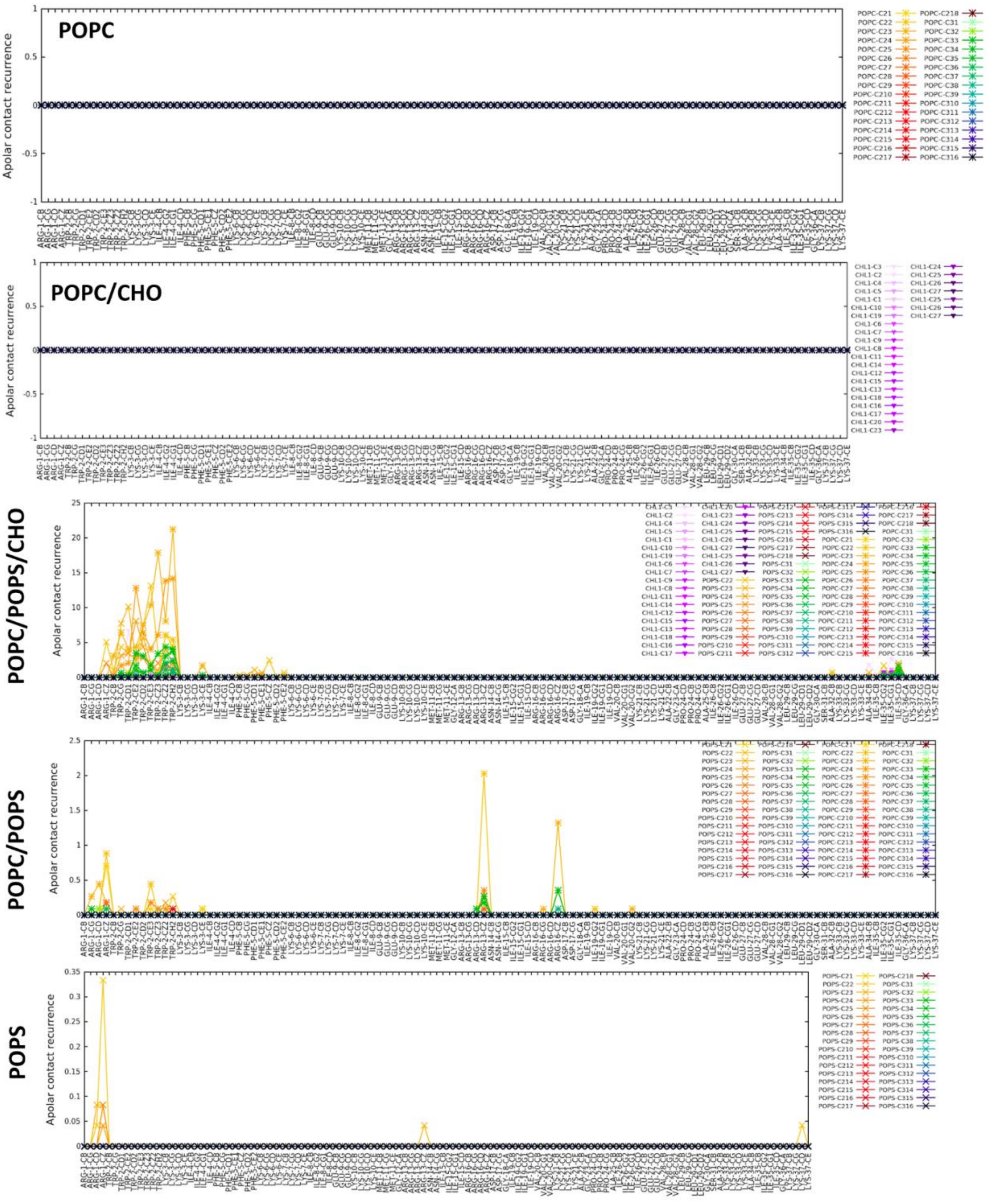

Figure S5. Occurence of van der Waals contacts between CXJ peptide and various membrane bilayers calculated along MD simulation trajectories.

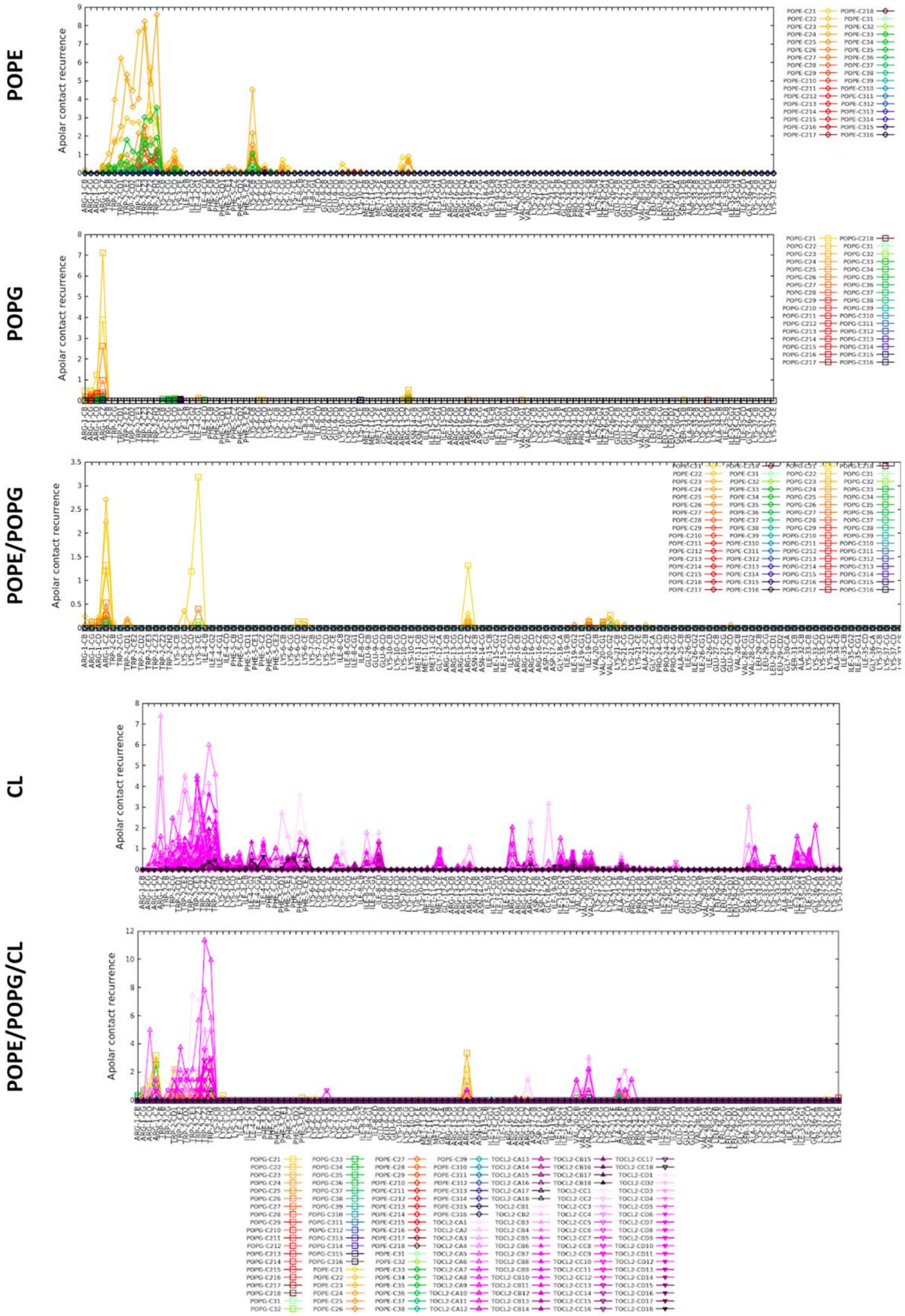

Figure S6. Occurence of van der Waals contacts between CXJ peptide and various membrane bilayers calculated along MD simulation trajectories. TOCL2 refers to CL.

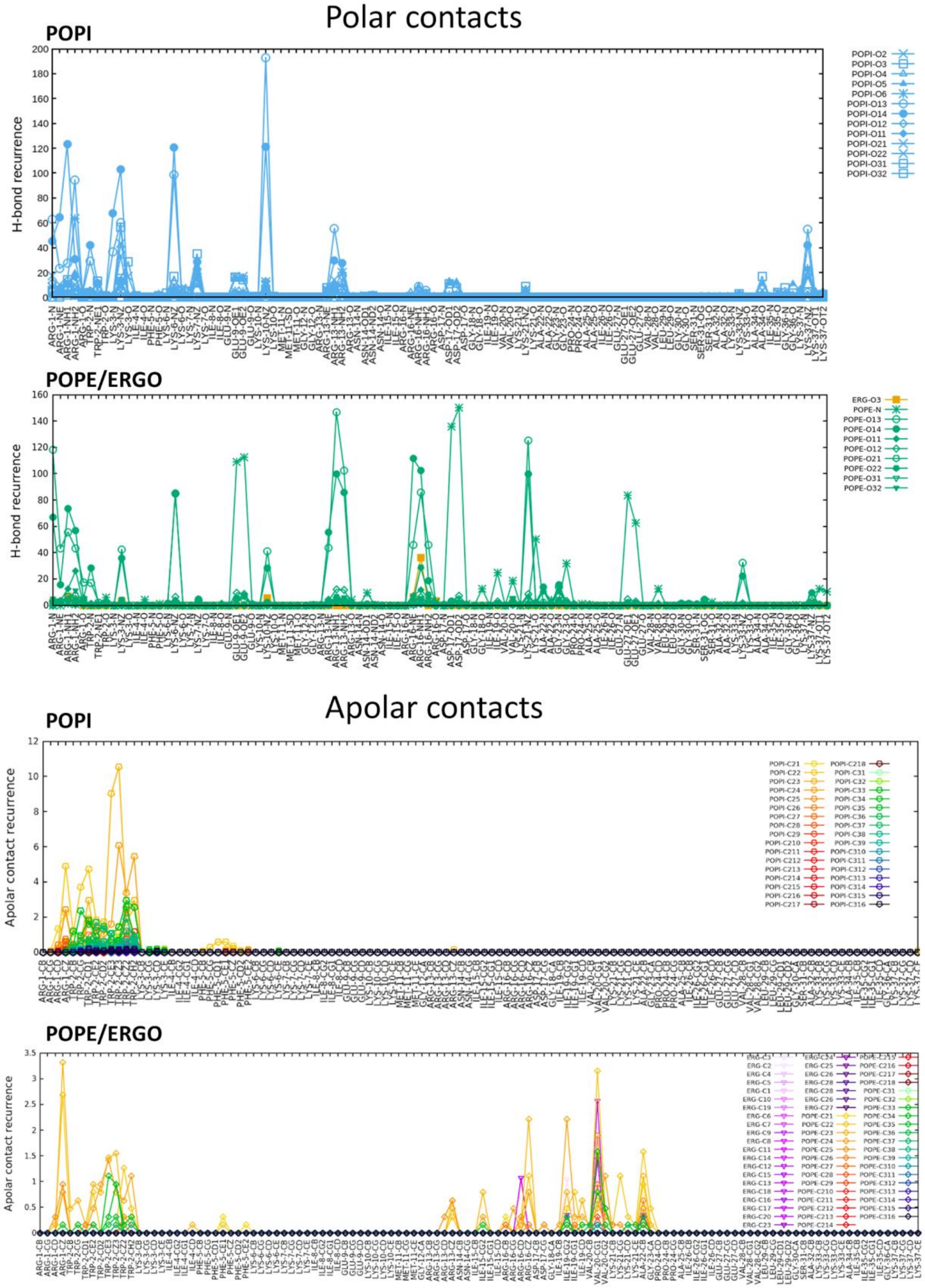

Figure S7. Occurrence of polar and apolar contacts with fungal-like membranes containing POPI and POPE/ERGO.

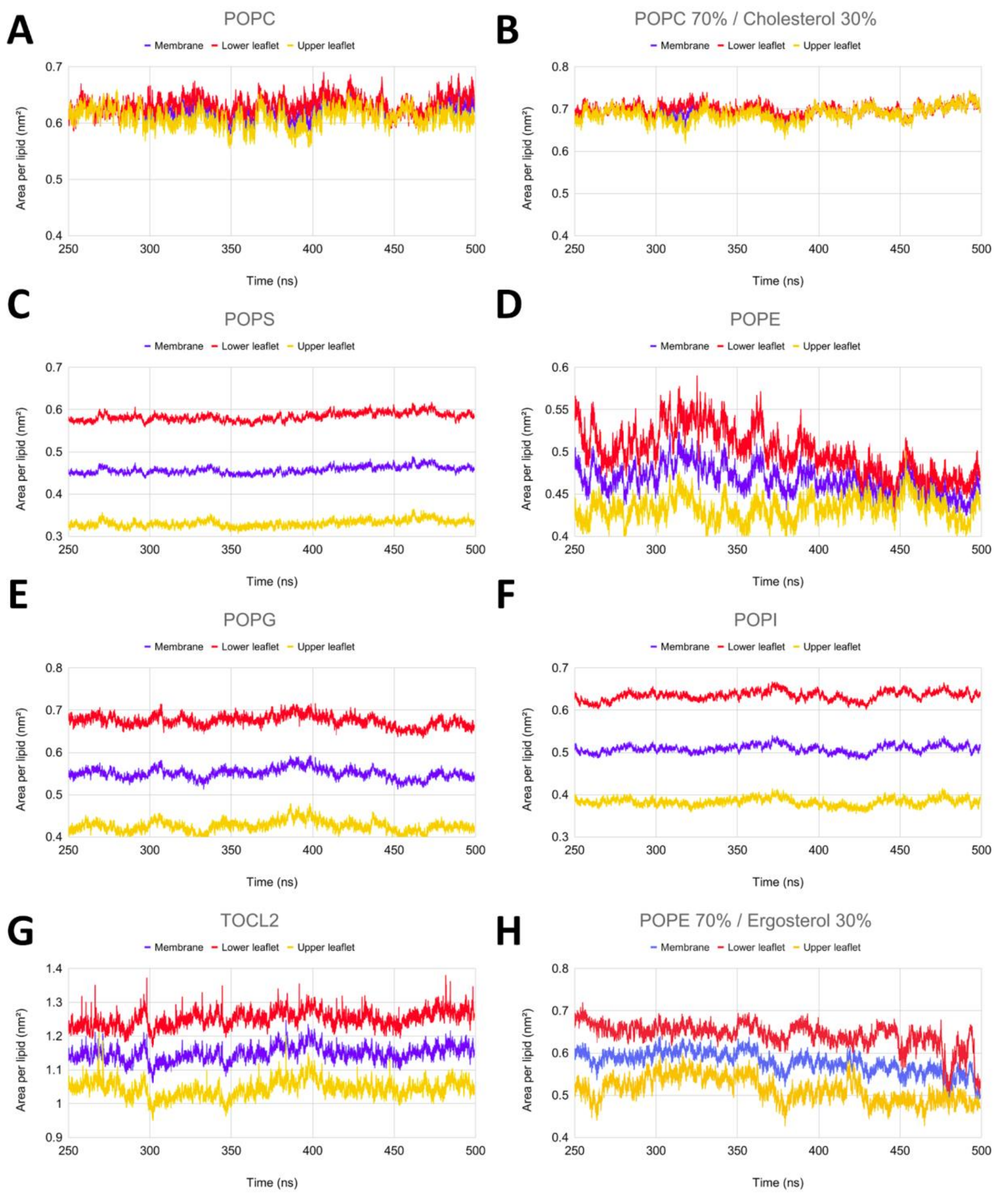

Figure S8. Area per lipid (nm^2) in bilayers containing various phospholipids compositions as calculated from MD simulations in the presence of eight CXJ peptides. The average value is shown in blue while the upper and lower leaflet are shown in yellow and red respectively. TOCL2 refers to CL.

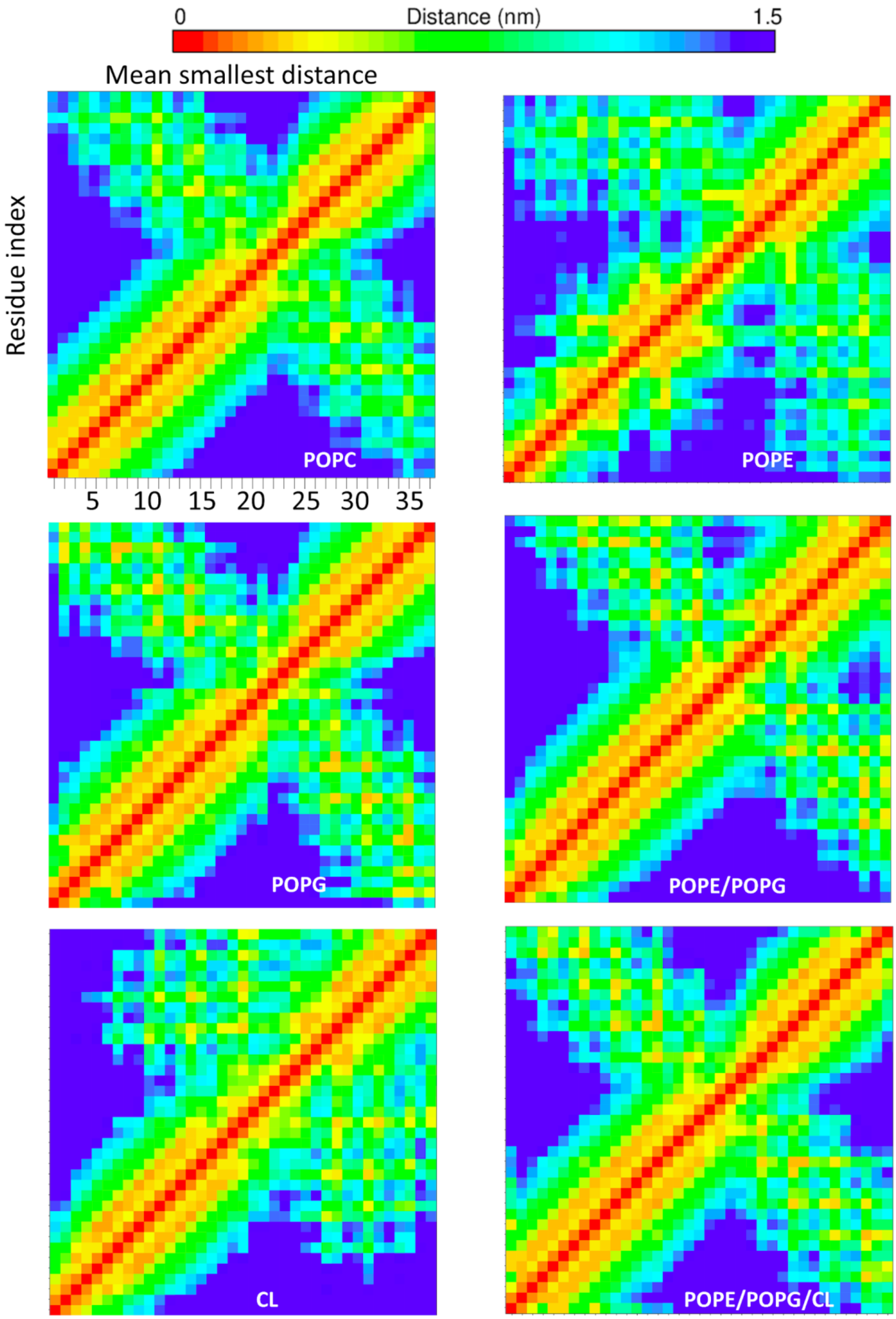

Figure S9. Contact maps comparing CXJN conformation with POPC/CHO, POPI, POPE/ERGO and POPS-containing membranes. TOCL2 refers to CL.

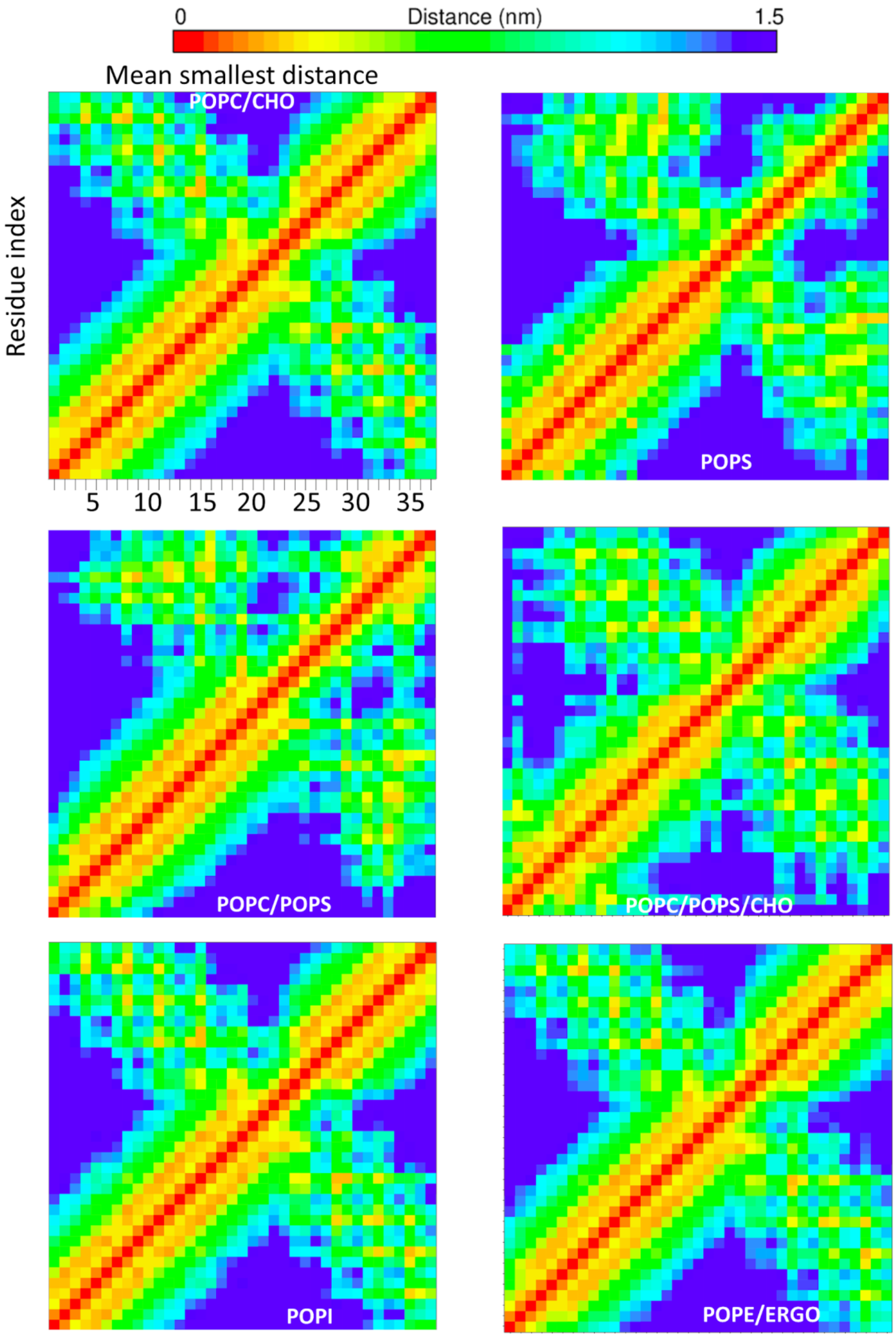

Figure S10. Contact maps comparing CXJN conformation with POPC/CHO, POPI, POPE/ERGO and POPS-containing membranes. TOCL2 refers to CL.

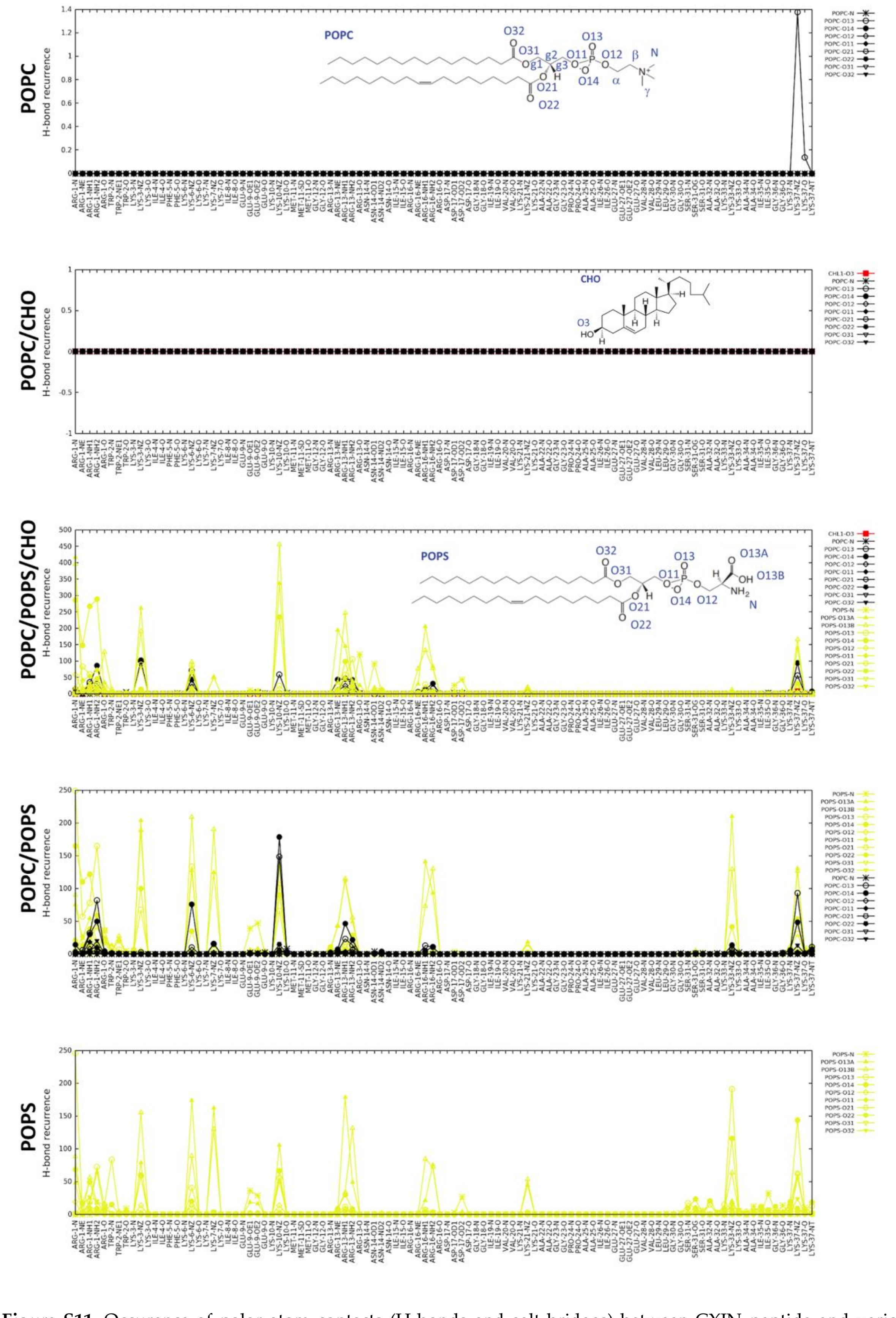

Figure S11. Occurence of polar atom contacts (H-bonds and salt bridges) between CXJN peptide and various membrane bilayers calculated along MD simulation trajectories.

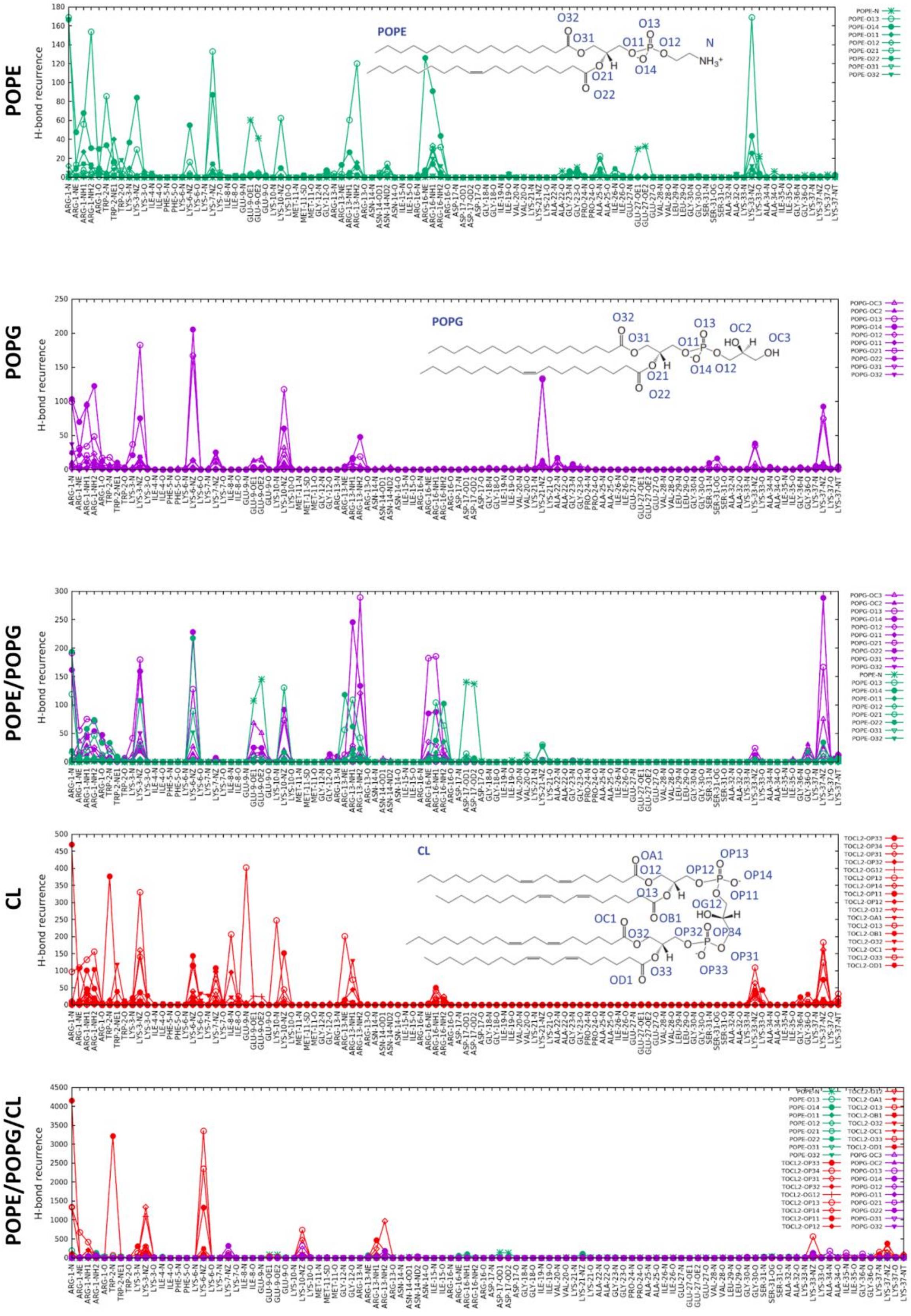

Figure S12. Occurence of polar atom contacts (H-bonds and salt bridges) between CXJN peptide and various membrane bilayers calculated along MD simulation trajectories. TOCL2 refers to CL.

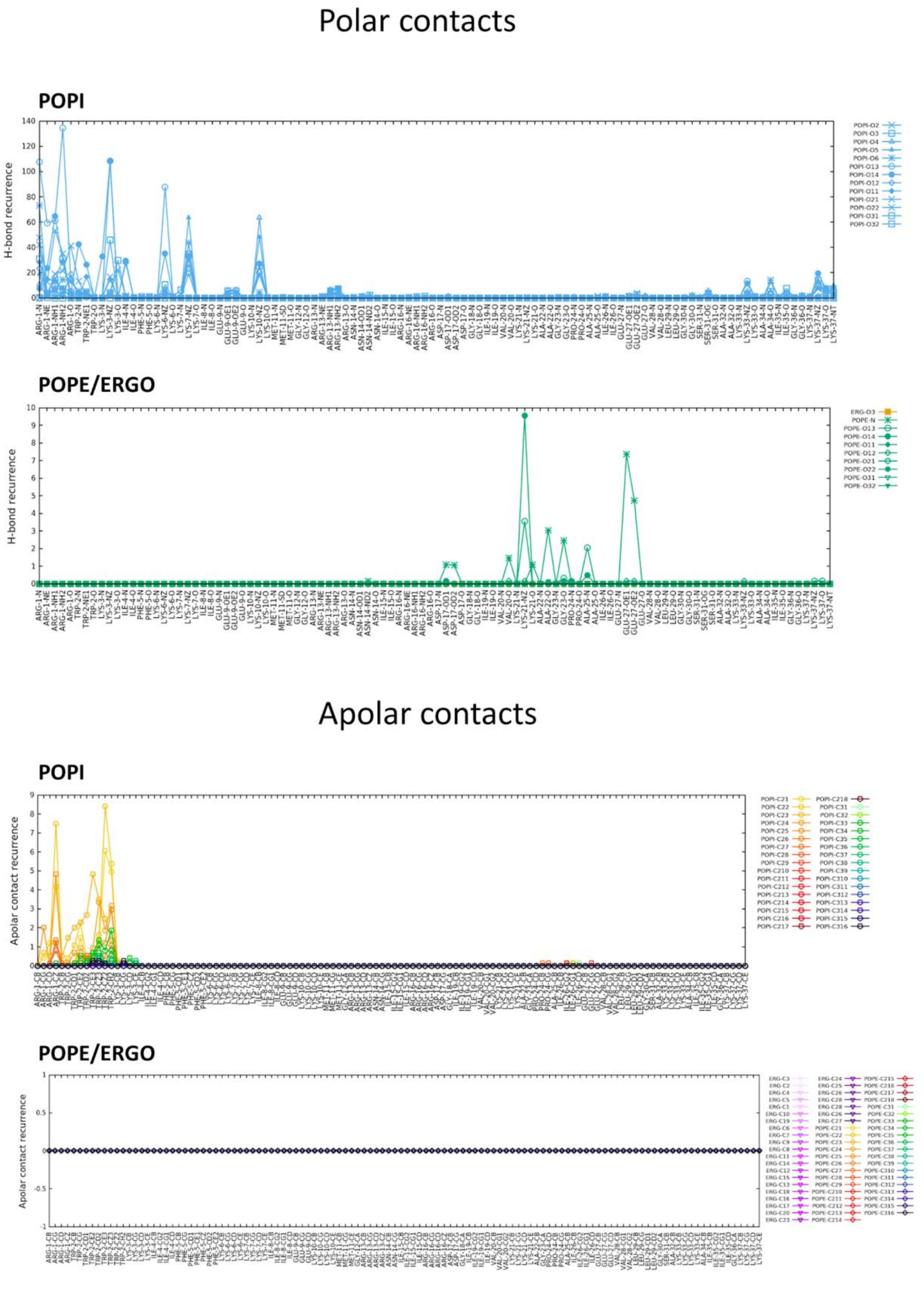

Figure S13. Occurrence of polar and apolar contacts between CXJN peptide and fungal-like membranes containing POPI and POPE/ERGO.

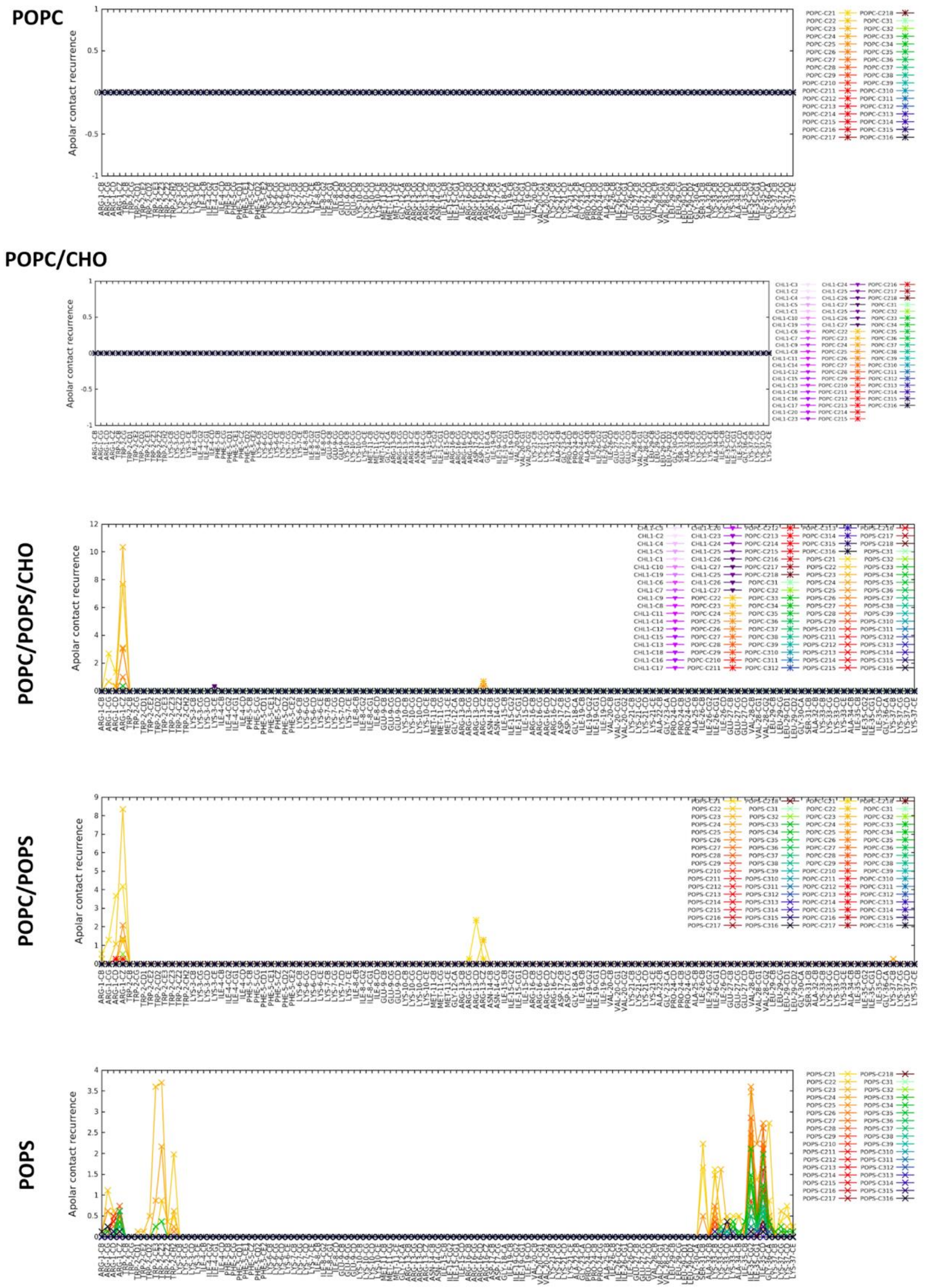

Figure S14. Occurence of van der Waals contacts between CXJN peptide and various membrane bilayers calculated along MD simulation trajectories.

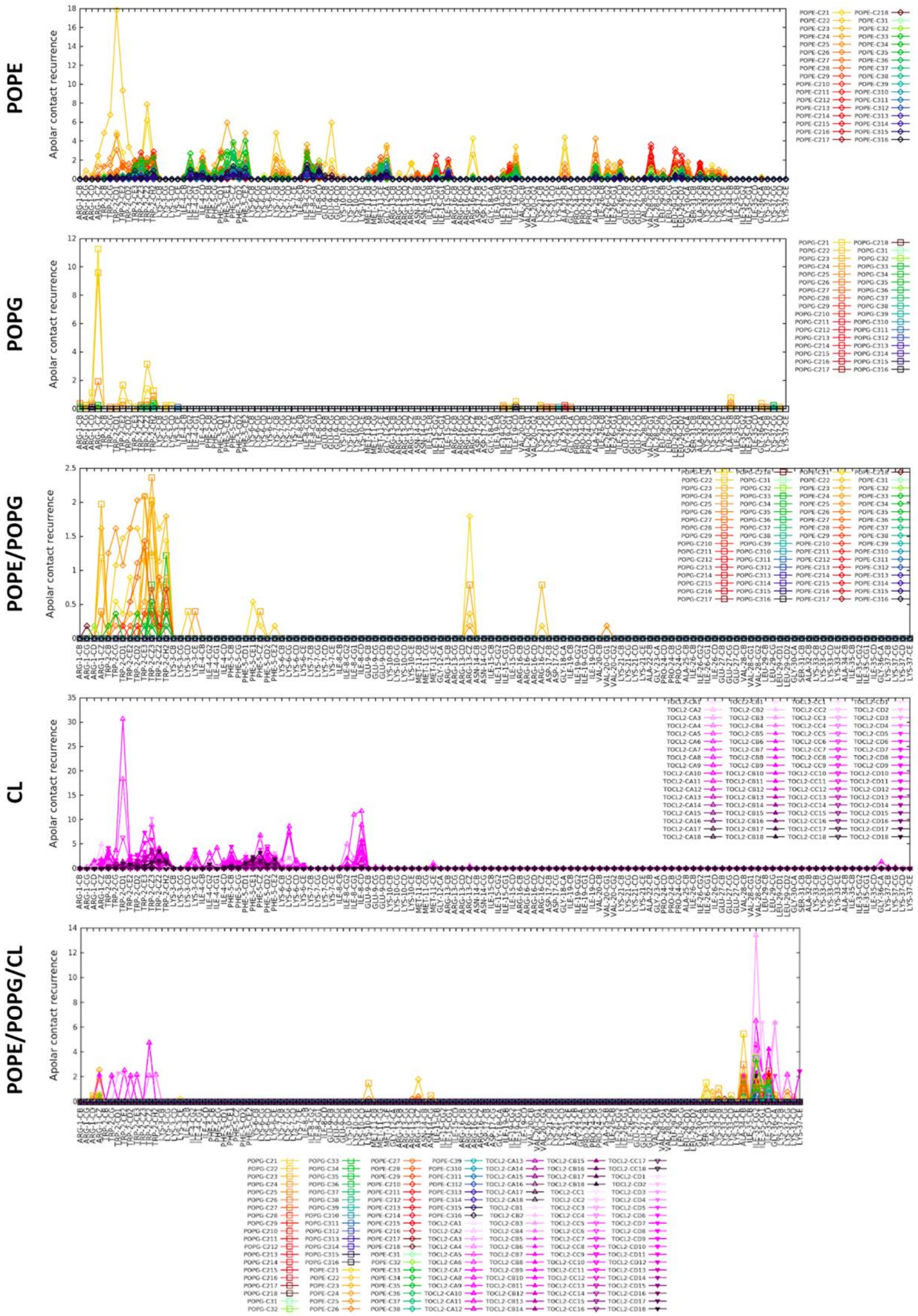

Figure S15. Occurence of van der Waals contacts between CXJN peptide and various membrane bilayers calculated along MD simulation trajectories. TOCL2 refers to CL.

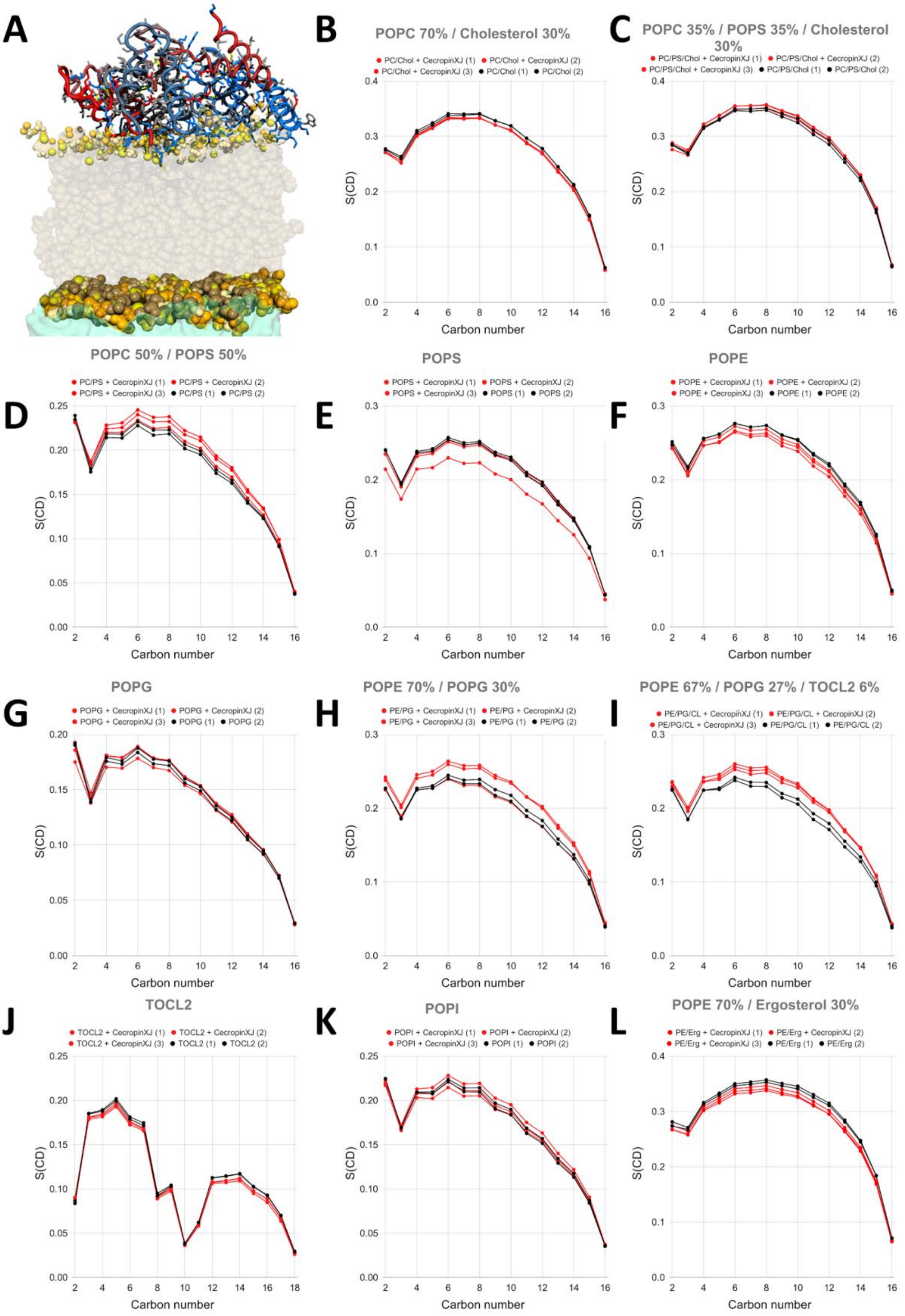

Figure S16. Order parameter of C-H moieties of palmitoyl side chains in membranes containing various phospholipids compositions as calculated from multiple repetitions of MD simulations in the absence (2 repetitions in black labeled as 1 and 2) and in the presence (3 repetitions in red labeled from 1 to 3) of eight CXJN peptides. The panel in the top left corner is an example of MD snapshot with POPS bilayer (color code in the caption of Figure 3). TOCL2 refers to CL.
